# Mammographic parasitic calcifications in South West Nigeria: prospective and descriptive study

**DOI:** 10.11604/pamj.2013.15.126.2958

**Published:** 2013-08-08

**Authors:** Adenike Temitayo Adeniji-Sofoluwe, Millicent Olubunmi Obajimi, Abideen Olayiwola Oluwasola, Temitope O Soyemi

**Affiliations:** 1 University of Ibadan, College of Medicine, Department of Radiology & University College Hospital, Ibadan, Nigeria; 2 University of Ibadan, College of Medicine, Department of Pathology & University College Hospital, Ibadan, Nigeria

**Keywords:** Parasite, mammography, calcification

## Abstract

**Introduction:**

Lymphatic filariasis caused by nematode parasite *Wuchereria bancrofti and Brugia Malayi* is endemic in the tropics. In Nigeria, 25% of the population is infected. Lymph edema and elephantiasis are the predominant manifestations. Its infrequent manifestation is in the breast. This paper discusses the epidemiology, reviews literature, imaging options and mammographic appearances of these parasitic nematodes.

**Methods:**

This prospective descriptive study reports on 39 cases of parasitic calcifications seen during mammography in the Radiology Department, University College Hospital between 2006 and 2012 in Ibadan, South West Nigeria. Each mammogram was reported by MO and ATS: assigned a final Bi-RADs category. Parasitic calcifications were further evaluated for distribution, and types of calcification.

**Results:**

A total of 527 women had mammography done between 2006 and 2012. Thirty-nine women (7.4%) had parasitic breast calcifications. The ages of the women ranged between 38-71 years - mean of 52.36±8.72 SD. Twenty-three (59%) were post-menopausal, 16(41%) were pre-menopausal. The majority (31; 79.5%) were screeners while 8(20.5%) were follow up cases. Approximately half (51.3%) of the women had no complaints. Pain (23.1%) was the commonest presentation in the remaining half. Solitary calcifications were predominant (20) while only 3 cases had 10 calcifications. Left sided calcifications (53.8%) were the majority. Calcifications were subcutaneous in 2/3rds of the women (66.7%) while the Yoruba tribe (84.6%) was principal.

**Conclusion:**

Parasitic breast calcifications can be misdiagnosed on mammography for suspicious micro-calcification. This publication should alert radiologists in a tropical country like Nigeria to increase diagnostic vigilance thereby preventing unnecessary anxiety and invasive work-up procedures.

## Introduction

Filariasis is common in most tropical and subtropical countries [1]. In Nigeria, the epidemiology of the disease is complicated because of the multiplicity of the environmental conditions of the different regions [2]. The distribution is more extensive than has been described because of deteriorating drainage systems, large-scale dam and irrigation projects which have created suitable breeding sites for filarial vectors in various parts of Nigeria3. In the past six decades, various levels of endemicity of filariasis have been documented in different parts of Nigeria including Benue, Plateau, Taraba, Oyo, and Bauchi states [2–4]. One out of every three sufferers of filariasis in the tropics lives in the Federal Republic of Nigeria [5].

Lymphatic filariasis is caused by the nematode parasite Wuchereria bancrofti and Brugia malayi. Other studies show filariasis due to Mansonella perstans and Mansonella streptocerca infections vastly recorded in different parts of Nigeria [6]. It is endemic throughout the tropics [7]. It is equally distributed in both sexes [8] and affects an estimated 120 million people worldwide7. In Nigeria as much as 25% of the population are infected [9]. The predominant manifestations of the infection are caused by obstruction of the lymphatics which leads to lymphedema, hydrocele and elephantiasis. An infrequent manifestation is lymphatic filariasis involving the female breast [10–13].

This study of mammographic findings in 39 women in Ibadan South West Nigeria with parasitic calcification of the breast is aimed at increasing the diagnostic vigilance for the parasites thereby preventing misdiagnosis for suspicious micro-calcifications in a country that is endemic for the disease.

## Methods

A prospective descriptive study carried out in the Radiology Department of the University College Hospital between 2006 and 2012. Thirty-nine (39) women were involved, out of 527 women who presented for both screening and diagnostic mammography. Informed consent was obtained from the identified women; after which an assisted questionnaire was administered before a physical examination was performed. Two standard views MLO and CC were obtained utilizing the General Electric senographe 2000 machine. Additional Spot magnification views were also obtained for evaluation of these calcifications. MO and AA reported these mammograms and assigned a final Bi-RADs category. Details of distribution and types of calcification as well as demographics of the women with the parasitic calcifications were described. Breast ultrasound scan was additionally performed with a linear-array, 10 MHz transducer.

## Results

A total of 527 women had mammography done between 2006-2012. Thirty-nine women (7.4%) had parasitic calcifications in their breast. The ages of the women ranged between 38-71years with a mean of 52.36(SD=8.725). Twenty-three (59%) were post-menopausal while 16(41%) women were pre-menopausal.

The majority 31 (79.5%) were screeners while 8(20.5%) were follow up cases. About half of the women (51.3%) had no complaints at presentation. However pain (23.1%) was the most common presentation in the remaining half.

**Figure 1:**
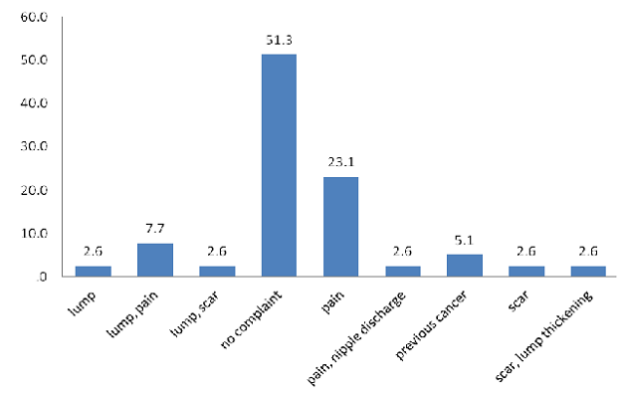
Presenting complaints of women

**Figure 2:**
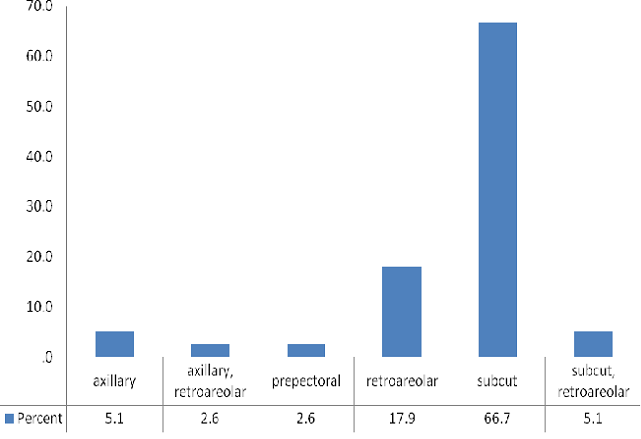
Location of calcifications

**Figure 3:**
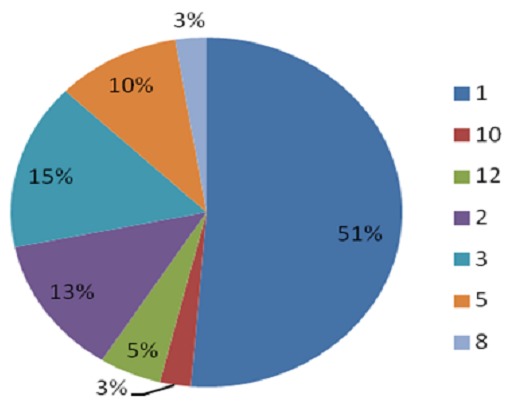
Number of parasitic calcifications

**Figure 4:**
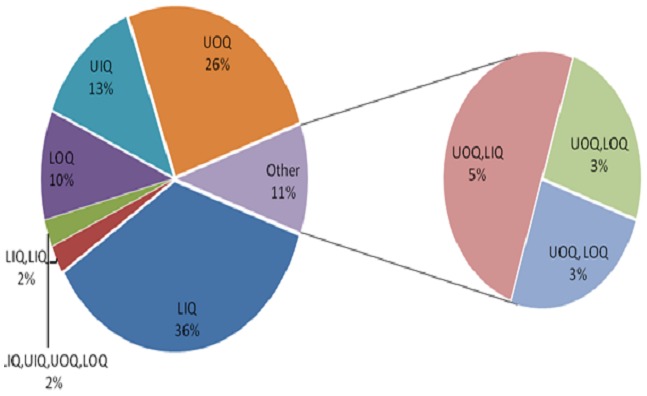
Quadrant of Breast Involved

**Figure 5:**
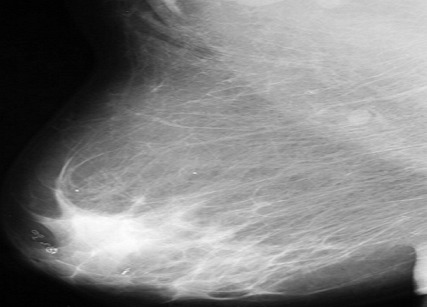
Patterns of parasitic calcifications seen at mammography on an MLO view

**Figure 6:**
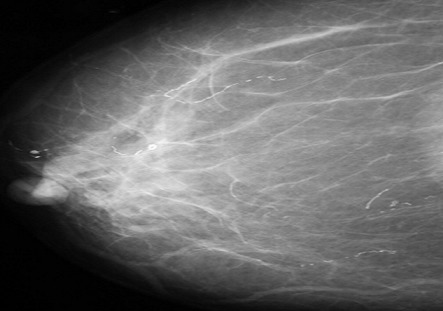
Patterns of parasitic calcifications seen at mammography on a CC view

**Figure 7:**
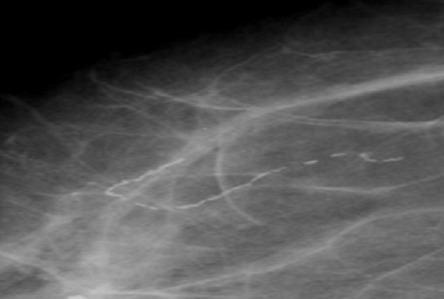
Spot Magnification Mammographic view showing linear, tubular parasitic calcifications

Solitary calcifications were present the majority (20) of cases while only 3 cases had more than 10 calcifications. Parasitic calcifications were most commonly left sided (53.8%) whereas both breasts were least frequently affected (12.8%). Most calcifications seen were located in the LIQ and UOQ (38.5% and 30.8% respectively). The four quadrants of the breast were involved in only one patient. Two-thirds (66.7%) of the parasitic calcifications were subcutaneous in location (Figure 1, Figure 2, Figure 3, Figure 4, Figure 5, Figure 6, Figure 7).

The women were predominantly of the Yoruba tribe (84.6%) and domiciled in Ibadan (74.5%). The size of the calcifications ranged between 1-4mm. Whole breast ultrasound was performed on all women but showed no abnormality.

## Discussion

Filariasis is uncommon in developed countries; where it is seen occasionally in travel immigrants from endemic areas of the disease [9, 14]. The prevalence of lymphatic filariasis is over 40 million in sub-Saharan African accounting for about 40% of all cases worldwide [15]. It is endemic in the tropics where Nigeria is situated. In Oyo state, four major rivers flow through one of the local government areas in a north to south direction, the Ogun, Oyan, Ofiki and Opeki and these have been documented as *Simulium* breeding sites [16]. A detailed history revealed that the women are domiciled in Ogun, Ondo, Lagos and Ibadan which are in the South West geopolitical zone of Nigeria [17]. A history of previous community screening for the disease was elicited with endemicity of filarial disease in the towns of their nativity but no form of treatment for the disease was obtained from the women [3, 16].

Though peripheral blood film was negative for microfilaria in these women making clinical diagnosis difficult. However a negative peripheral blood smear does not preclude the diagnosis of filariasis. This was confirmed in a study conducted by Ogunba [18] in Nigerians infected with loa loa, with up to half of the cases with low peripheral microfilaria levels. Other techniques that may be useful for the diagnosis are tests for antifilarial antibodies/circulating parasitic antigens and polymerase chain reaction (PCR) which is expensive and not frequently performed in Nigeria [15]. No biopsy was performed in the patients.

Fredman and Kalisher [9], reported transmission of filariasis by means of a bite from a mosquito (that has previously bitten an infected host). The mosquito then deposits infective larvae on the skin. The larvae migrate through the puncture wound to reach the subcutaneous vessels where they mature into the adults over a period of 6-months to 2 years. The patient's infection can remain dormant for decades which are likely in these women. The adult worms mate and produce microfilaria which circulates in the blood stream with a periodicity that generally matches the feeding habits of the mosquito vector [14]. The life cycle is completed when the microfilaria are ingested by the mosquito. W. bancrofti the predominant variant of the two types is more common in Nigeria. The other variant B. Malayi is responsible for infection in parts of Asia [14]. The clinical presentation of the infection is due to lymphatic dysfunction, inflammation and obstruction. Later, this region drained by the local lymphatic system is prone to secondary bacteria and superficial fungal infection resulting in further lymphatic damage the so-called granulomatous *lymphangitis* and may lead to skin changes called elephantiasis [7, 9, 14].

The breast is a recognized though infrequent site of involvement [14]. In the breast, granulomatous lymphangitis develops in the tissue adjacent to the lymphatic vessel [11] and eventually block the vessels which are soon replaced with fibrous tissue [8] and the patient may present with a palpable breast mass, the filarial granuloma or mass and microcalcifications. In a review of 131 cases of filarial granuloma of the breast [11], it was reported that in most cases, the solitary and superficial filarial nodule was palpable and in more than half the cases the clinical impression was suggestive of a breast tumour, one patient even underwent a simple mastectomy. However, none of these patients presented with a palpable mass rather, their mammograms demonstrated tortuous, ring like microcalcifications like the report by Chow et al [14]. The above typical mammographic appearance and endemicity of the disease supported the diagnosis of filariasis. Filarial calcifications of the breast can be found anywhere in the breast including the nipple and the retroareolar region [8]. This is reiterated in our patients, where calcifications were found in virtually all the quadrants within the breast parenchyma as well as in the retroareolar region.

However, their mammographic features differ from the nematodal infections caused by *Onchocerca volvulus (Onchoceriasis), loa loa* and *Trichinella spiralis (trichinosis)* [19]. The filarial infection of O.volvulus and Loa loa are distinguished from the infection of W.bancrofti on the basis of the location and appearance of the organisms. While Wuchereria bancrofti reside in the breast parenchyma, onchocerca volvulus organisms are found in spaces just beneath the skin epithelium and occasionally in lymphatic vessels and lymph nodes where their calcifications form intricate tangles [8]. The calcifications of loiasis differ from those of W. bancrofti on the basis of their appearance as either short or long continuous or beaded fine calcifications, when the worm is extended. Loiasis may also appear as hair-like whorls of calcifications. Peculiarly, *Trichinosis* calcifications are located only in the pectoral muscles, they are however much smaller, more numerous and not serpiginous when compared with those of filariasis [20].

Records show that the skin over the parasitic nodule may be hyperaemic with changes of *peau d'orange* and axillary nodal enlargement which may mimic a suspicious breast lesion. However, in a multinational and multicenter study carried out in 1997 in Oyo state -Nigeria, a nodule prevalence rate of 59% was found in the three Ibarapa local government areas in the state, but [21] none of these women had nodules or hyperaemia.

These skin and axillary changes are known features of breast cancer and could lead to a misdiagnosis in our setting where mammographic breast imaging is in its infancy. These suspicious features are then over treated as reported by Chen [10] more so in a developing country that lacks the full complement of further comprehensive breast work-up with Magnetic Resonance Imaging (MRI), stereotactic biopsy and needle localization.

On the overall, benign dystrophic calcifications common in the breast can also be differentiated from those of parasitic diseases on the bases of their coarse, dense and irregular appearance [8].

Chen and Xie [10] also warned that foci of calcification of worm are frequent presentation rather than the occasional calcifications of the whole and entire worms. These foci of calcifications could simulate suspicious comedo calcifications and could be wrongly treated as a cancer. In this instance, it is recommended that other stigmata of cancer be considered before a final diagnosis is made. Previously, the full worm calcifications were considered intermediate; which led to localization and excision biopsy [14]. Now with increased awareness of the disease, diagnosis is likely to be more accurate as other ancillary investigative tools could be utilized for final diagnosis. Recent advances in breast Imaging has made sonomammography invaluable adjunct in the evaluation of breast disease considering its usefulness in the evaluation of the axilla. There is however a dearth of literature of this imaging tool in the assessment of filarial disease. Evaluation of the breasts with sonography confirmed no definite mass and demonstrated no calcifications, reaffirming the latter's insensitivity in the evaluation of calcifications in breast disease and making mammography [22] the gold standard for screening.

One of the complications of excisional biopsy is the appearance of residual calcifications in follow-up mammograms. The residual micro-calcifications of degenerating and calcifying nematodes may also be confused with malignant calcification [14].

It is therefore important that filarial calcifications are critically assessed, diagnosed accurately and properly categorized to avoid an unnecessary, expensive, invasive procedure like excision biopsy.

## Conclusion

This paper illustrates that wormlike calcifications are also visible on mammograms. Radiologists should be aware of the endemic nature of filariasis in the country. As routine mammography becomes available in Nigeria, there is a need for a high level of suspicion for parasitic calcifications in the breast parenchyma in mammographic images so that the infection will not be misdiagnosed for a malignant breast lesion.
